# The performance of a machine learning model in predicting accelerometer-derived walking speed

**DOI:** 10.1016/j.heliyon.2025.e42185

**Published:** 2025-01-22

**Authors:** Aleksej Logacjov, Tonje Pedersen Ludvigsen, Kerstin Bach, Atle Kongsvold, Mats Flaaten, Tom Ivar Lund Nilsen, Paul Jarle Mork

**Affiliations:** a Department of Computer Science, Norwegian University of Science and Technology (NTNU), Trondheim, Norway; b Department of Public Health and Nursing, Norwegian University of Science and Technology (NTNU), Trondheim, Norway

**Keywords:** Epidemiology, Validity, Physical activity

## Abstract

**Background:**

Obtaining long-term measurements of walking speed in large-scale studies remains challenging. The aim of this study was to develop and evaluate the performance of a machine learning classifier in predicting slow (≤4 km/h), moderate (4.1–5.4 km/h), and brisk (≥5.5 km/h) walking speeds in adults based on dual and single accelerometer set-ups.

**Methods:**

Twenty-four adults (mean age [SD, range] 36.1 [11.9, 23–62] years) participated in the study. Two tri-axial accelerometers positioned on the thigh and low back were used to record body movements. A measuring wheel with a speedometer along with video recording were used to define and record consecutive 5-min periods with the three walking speeds and jogging during conditions resembling free-living. In addition, we included a 5-min period with gradual increase and decrease in walking speed from slow to brisk and vice versa. The video recordings were labelled and used as ground truth for training an eXtreme Gradient Boosting (XGBoost) machine learning classifier. Windows of 1, 3, and 5 s duration were used to train the classifier. The performance of the classifier was evaluated by leave-one-out cross-validation.

**Results:**

Total recording time was ∼600 min (∼25 min per participant). Performance metrics for predicting walking speeds (i.e., slow, moderate, brisk) and jogging were largely similar for the dual and single accelerometer set-ups as well as for the different window lengths. The highest overall accuracy was 91 % (SD 11 %, range 59–98 % for individual participants) using a dual accelerometer set-up and a 5-s window, whereas the lowest overall accuracy was 88 % (SD 11 %, range 51–96 % for individual participants) using a single thigh accelerometer set-up and a 1-s window.

**Conclusions:**

A machine learning classifier can be used to accurately predict slow, moderate, and brisk walking speeds based on both a dual and single accelerometer set-up on the thigh and/or low back.

## Highlights

• Long-term measurements of walking speed remain a challenge.
• Real-life walking speed can be accurately predicted based on accelerometer data.
• This allows further investigation into the impact of walking speed on health.

## 1 Introduction

Walking speed is a relevant parameter for evaluating changes in health status and predicting health outcomes [1,2]. Numerous studies have demonstrated a robust association between a slow usual walking speed and reduced physical function and increased mortality [[3], [4], [5], [6], [7], [8]]. Conversely, a higher usual walking speed is associated with substantial health benefits [9].

In large-scale population-based studies, the assessment of walking speed most often relies on self-report [[9], [10], [11], [12]], which is susceptible to measurement error and bias [13]. Technological advancements in device-based measurements of physical activity in population-based studies provide an opportunity for prolonged, real-world measurements of walking speed [[14], [15], [16]]. In contrast to standardized measurements conducted in controlled settings, such as clinical facilities, long-term measurements of usual walking speed offer higher ecological validity and can give valuable insights into health benefits associated with volume of different walking speeds.

Recent studies indicate that instantaneous walking speed can be estimated with reasonable accuracy based on wrist-worn accelerometry [17,18], arrays of skin-mounted accelerometers on the sacrum and lower limbs [19], and a single accelerometer on the trunk or low back [18,20]. However, other widely adopted accelerometer set-ups in population-based studies, such as a single thigh accelerometer [21,22] or a combination of thigh and low back [16], lack evaluations for predicting walking speed. These set-ups have proven effective in accurately predicting modes of physical activity and postures using an eXtreme Gradient Boosting (XGBoost) machine learning classifier [23,24]. However, the ability to predict walking speed based on a single or dual accelerometer set-up on the thigh and low back remains unexplored.

The aim of the current study was to develop and assess the performance of a machine learning classifier in predicting slow (i.e., <4 km/h), moderate (4.1–5.4 km/h), and brisk (>5.5 km/h) walking and jogging (>6.4 km/h) based on features extracted from accelerometry measured at the thigh and low back.

## 2 Material and methods

### 2.1 Measurement protocol and instrumentation

Twenty-four healthy adults (14 females, 10 males) were recruited among university/hospital staff to participate in the current study on walking speed (Table 1). The recruitment of participants was purposeful to ensure that the age span, gender, and anthropometrics varied sufficiently to approximate a healthy adult population. The Regional Committee for Ethics in Medical Research, Mid-Norway, evaluated the study protocol. Since the study does not include health data or personal data the decision was that the study does not require ethical approval (reference no. 2015/1432).

**Table 1:** Characteristics of the study sample. Values are mean (SD), range.

Variable	Females (n = 14)	Males (n = 10)
Age, years	35.5 (12.1), 23-60	36.9 (12.2), 26-62
Weight, kg	65.7 (8.6), 58.4–91.0	78.6 (8.8), 67.4–99.5
Height, cm	170.0 (7.5), 156.5–188.0	183.9 (8.0), 173.0–198.5
Body mass index, kg/m^2^	22.8 (3.0), 20.7–30.0	23.2 (1.7), 19.9–26.7

The procedure for collecting the accelerometry data has been described in detail elsewhere [23,24]. In short, participants were equipped with two small lightweight tri-axial AX3 accelerometers (23 × 32.5 × 7.6 mm, weight 11 g; Axivity Ltd., Newcastle, UK) positioned at the front of the right thigh (approximately 10 cm above the upper border of the patella) and centrally on the lower back at the third lumbar vertebra (L3). A 5 × 7 cm moisture permeable film (Opsite Flexifix; Smith & Nephew, Watford, UK) was attached to the skin and the sensors were then positioned on top of the film using double-side tape and covered with a second film layer of 10 × 8 cm. The accelerometers were configured to record at 50 Hz and a range of ±8 g. The raw data was stored on a 512 MB internal memory card and downloaded as binary file in the Continuous Wave Accelerometer (CWA) format for visualization and further analysis. Further technical details are available at https://github.com/openmovementproject/openmovement/blob/master/Docs/ax3/ax3-technical.md.

A measuring wheel (Blinken, Model 1176, Blinken AS, Norway) with a speedometer (CatEye Padrone CC-PA 100WA, Japan) and a video camera (GoPro Hero 3+, US) was used to define and measure the different walking speeds and jogging. The video camera was positioned to record the speedometer display and the lower body of the participants while walking and jogging. To record and control walking speed, a research assistant followed directly behind the participant with the measuring wheel. If necessary, the assistant provided feedback to the participant whether to increase or decrease walking/jogging speed to stay within the target speed range. The protocol was performed in walkways at a university hospital.

The participants were instructed to walk at a low (≤4 km/h ∼ ≤1.11 m/s), moderate (4.1–5.4 km/h ∼ 1.12–1.50 m/s), and brisk (≥5.5 km/h ∼ ≥1.51 m/s) walking speed in consecutive 5-min periods. These cut-off values were chosen based on the compendium of physical activities, resembling walking intensities categorized as light, moderate, and brisk [25,26]. Jogging (>6.4 km/h ∼ >1.8 m/s) was included to examine whether the classifier could discriminate between brisk walking and jogging. Following the compendium coding scheme, our categories of walking speed approximate ≤3.0 metabolic equivalents of task (METs; slow, code 17170), 3.1–4.2 METs (moderate, code 17190), and ≥4.3 METs (brisk, code 17200), respectively. Similar as for the different walking speeds, the period with jogging lasted 5 min, approximating an intensity of 6.0 METs (code 12029) [25]. In addition, we included a 5-min bout with a gradual increase in walking speed from slow to brisk and thereafter a gradual decrease back to slow walking.

The participant’s exertion during the different walking speeds and running was assessed by heart rate (HR) and Borg’s scale of perceived exertion [27]. HR was recorded continuously throughout the measurements, using a Polar H9 chest strap connected to a Polar M400 GPS Smart Sports Watchsport (Polar Electro Oy, Kempele, Finland). HR data was downloaded to Polar Flow software for further analysis. Maximal HR (HRmax) was estimated using the method described by Tanaka and co-workers, i.e., 208–0.7 x age [28]. The relative exertion was calculated as %HRmax, using the average HR during the last 30 s for each of the 5-min periods with different walking speeds and running. The Borg’s scale ranges from 6 (no exertion) to 20 (maximal exertion) and participants were asked to rate their perceived exertion during each period within the different walking speeds and the period with jogging.

### 2.2 Training of the machine learning classifier

Recent studies have shown that a XGBoost machine learning classifier can predict key postures and physical activity types (including walking) with similar or higher overall accuracy as other machine learning classifiers [23,24]. The current study expands on the existing classification of walking. In short, the videos were downloaded from the memory card and converted into an AVI file format maintaining a frame rate of 25 fps. Using the Anvil software package [29], the videos were labelled frame-by-frame for the three target walking speeds and jogging. Prior to training the machine learning classifier, the accelerometer data from each sensor was exported from the raw files, resampled, normalized, and synchronized. The data streams (i.e., x-, y-, and z-axis) from the accelerometers and the video recording were synchronized by three heel drops at the beginning and end of the recording that were visible both in the accelerometer signal and video recording. The labelled video data was used as ground truth to train the XGBoost classifier [30].

The accelerometer signals were segmented into non-overlapping windows of 1, 3, and 5 s. For each window, 161 different statistical features were computed. A detailed description of the feature extraction has been provided in related publications [23,24]. A six-fold cross-validation with grid search was performed to find the optimal hyperparameters for the XGBoost model. The best hyperparameters were used for the final leave-one-subject-out cross-validation. We compared the different window sizes (i.e., 1, 3, and 5 s), as well as single and dual accelerometer set-ups (thigh, back, combination of thigh and back). All analyses were performed using Python 3.8.10 (with package versions: numpy 1.21.4; pandas1.3.4; scikit-learn 1.0.2; scipy 1.7.2; xgboost 1.5.0).

**Fig. 1:**
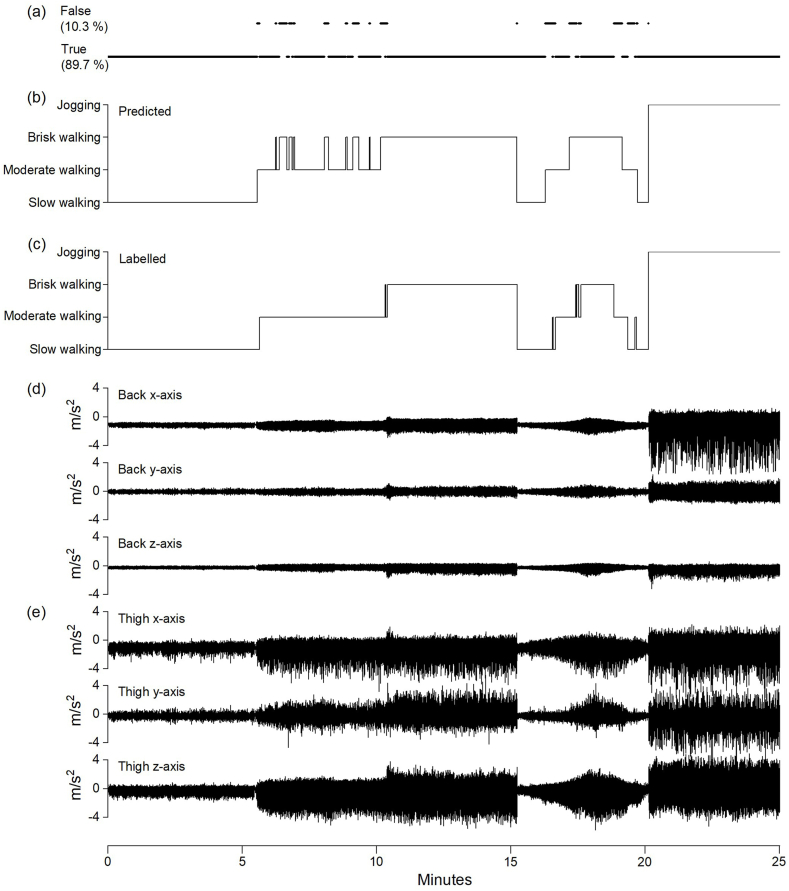
Example of a 25 min recording from one participant. The panels show the instances with true and false predictions (a), the predicted (b) and labelled (c) walking speeds and jogging using a 5 s window size, and the accelerometer recordings from the thigh (e) and low back (d).

Fig. 1 illustrates the protocol and shows an example of accelerometer recordings from the thigh and low back for one participant, the labelled walking speeds and jogging based on the video recordings (i.e., “ground truth”), the predictions produced by the XGBoost classifier, and the instances with true and false predictions.

### 2.3 Statistics and performance metrics

The predictive performance of the XGBoost classifier was evaluated using leave-one-out cross validation, i.e., the classifier is trained on the data from all participants except one, which is kept out and used as the test data set. Performance metrics included sensitivity, specificity, the F1 score, and accuracy. Sensitivity is the proportion of correctly identified true positives, calculated as the number of true positives divided by the sum of true positives and false negatives. Specificity is the proportion of correctly identified true negatives, calculated as the number of true negatives divided by the sum of true negatives and false positives. The F1 score is the harmonic mean of sensitivity and precision while accuracy is the proportion of correctly classified instances divided by the total number of samples. The result of each of these performance metrics is a value between 0 and 1 with a higher number indicating better performance of the classifier.

## 3 Results

Movement speed, %HRmax, and perceived exertion during the three walking speed conditions and jogging are presented in Table 2. As indicated by the min/max values, there was no overlap in movement speed between the walking speed or jogging conditions. The corresponding average %HRmax and perceived exertion increased markedly from slow walking to jogging, but there was considerable inter-individual variation.

**Table 2:** Movement speed (km/h), heart rate (%HRmax), and rate of perceived exertion during the three walking conditions and jogging. Values are mean (SD), range.

	Speed (km/h)	%HRmax	RPE
Slow walking	3.1 (0.4), 2.3–3.7	46.4 (5.2), 35.1–56.1	6.4 (0.5), 6-7
Moderate walking	4.9 (0.2), 4.4–5.3	49.7 (4.6) 39.9–56.4	7.6 (1.2), 6-10
Brisk walking	6.1 (0.3), 5.7–6.8	55.3 (5.2) 43.1–64.0	9.8 (1.7), 7-13
Jogging	8.3 (0.7), 7.1–9.7	72.0 (8.9), 49.2–86.4	12.2 (1.4), 10-15

Abbreviations: HR, heart rate; RPE, rate of perceived exertion.

**Fig. 2:**
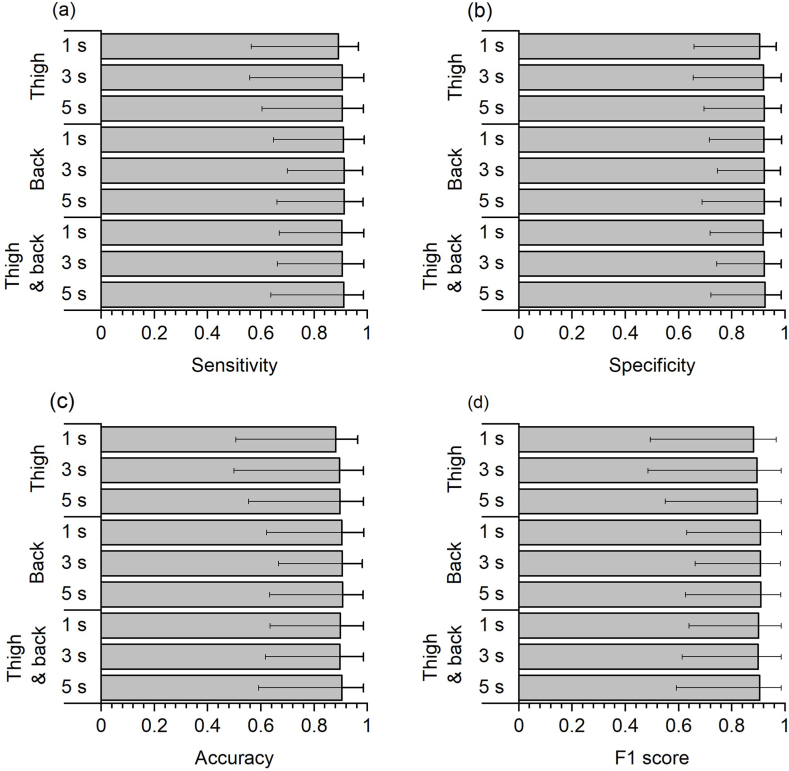
Performance metrics of the machine learning classifier in predicting the three walking speeds (slow, moderate, and brisk) and jogging for the different accelerometer set-ups and window lengths. Bars indicate mean values while error bars indicate range of individual values.

Fig. 2 shows the performance metrics for the machine learning classifier across all walking speeds and jogging for the different accelerometer set-ups and window lengths (see also tabular form in Supplementary Material 1). The average performance metrics were largely similar for the different accelerometer set-ups and window lengths, e.g., the lowest averaged accuracy was 88 % (SD 11 %, range 51–96 % for individual participants) based on the single thigh accelerometer set-up and 1 s windowing while highest averaged accuracy was 91 % (SD 11 %, range 59–98 % for individual participants) based on the dual accelerometer set-up and 5 s windowing (Fig. 1C and Supplementary Material 1). When considering the range for individual participants, there was a tendency of a somewhat wider lower range for the single thigh accelerometer set-up across the different performance metrics, especially for the 1 s and 3 s windowing.

**Fig. 3:**
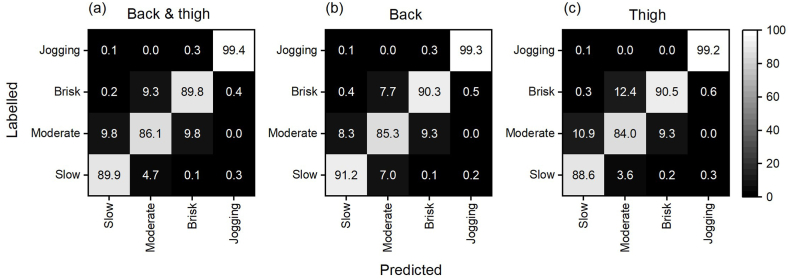
Confusion matrixes for the dual (a) and single accelerometer set-ups (b and c) based on classification using 5-s windows. The labelled walking speeds and jogging are shown in the rows, while the predicted walking speeds and jogging are shown in the columns. Values are row percentages. The bar to the right of (c) indicates the shading according to percentages.

Fig. 3 shows the confusion matrixes for the dual (a) and single (b and c) accelerometer set-ups using 5 s windowing. In general, misclassifications were more pronounced between the three walking speeds than between any of the walking speeds and jogging. For the single thigh accelerometer set-up, moderate walking was misclassified as brisk walking in 12.4 % of the instances. For the dual accelerometer set-up and the single low back accelerometer set-up, misclassifications were <9.8 % for all three walking speeds. In comparison, <0.3 % of the instances labelled as jogging were misclassified as walking for any of the accelerometer set-ups.

## 4 Discussion

The machine learning classifier developed in the current study demonstrated predicted slow, moderate, and brisk walking speeds with ∼90 % overall accuracy based on both a single and dual accelerometer set-up using a semi-structured measurement protocol, although the inter-individual prediction accuracy varied somewhat. Furthermore, the classifier separated jogging from walking with very high accuracy. The data used for training the classifier and the parameters for configuring the classifier are publicly accessible at https://github.com/ntnu-ai-lab/harth-ml-experiments. The approach described in the current study can therefore be adopted and further developed by other studies where thigh- and/or low back-worn accelerometry is collected and assessment of walking speed is relevant.

Recent studies have shown that key postures (lying down, sitting, standing) and physical activity types (walking, running, cycling) during free living can be predicted with high accuracy based on dual accelerometry at the thigh and low back, using a machine learning classifier [23,24]. The HARTH dataset used to train the classifier and the classifier itself is publicly available [24]. The current study expanded the machine learning classifier to detect broad categories of walking speed. Thus, the current model can be added as an additional post-processing level to the initial model [23] where periods detected as walking are further classified into the three walking speeds. Similar to the previous studies classifying key postures and physical activity types, the current results indicate that using 5-sec windowing for extracting information from the accelerometer signal leads to somewhat better results than using 1-sec or 3-sec windows. This is important since the computational cost in analyzing data is lower for longer window lengths.

A previous study showed that a single accelerometer on the thigh or low back is sufficient to predict key physical activity types (i.e., walking, running, and cycling), but performance was poor in discriminating between postures (i.e., standing, sitting, and lying) [23]. Notably, the performance of the machine learning classifier in the current study was largely similar for the single versus dual accelerometer set-ups, and for different window durations used for training the classifier. Thus, if applying the current expanded classifier and the study focus is on physical activity types (including walking speed) rather than on postures and/or sedentary behavior, it is sufficient to apply a single accelerometer on either the thigh or low back. This is in line with a recent study showing that treadmill walking speed can be predicted with reasonable accuracy from a single accelerometer at the thigh or sacrum, using a machine learning approach [19].

A dual accelerometer set-up as used in the current study allows an accurate prediction of key physical activity types and postures along with the prediction of walking [23,24]. Understanding how the interaction between interdependent physical activity types, postures, and sleep across 24-h affects health has been put forward as an important research priority [[31], [32], [33]]. In this context, interdependence implies that across 24 h, any increase in time allocated to one physical activity or posture must be offset by a decrease in at least one other physical activity or posture [34]. However, studies using a 24-h analytic approach have so far only considered walking as one single physical activity type. Since walking is a common form of exercise among adults, it is relevant to examine whether different time distribution between slow, moderate, and brisk walking yields dissimilar health benefits. Moreover, walking speed is an important indicator of health and health behavior and the current study contributes to bring the field forward by offering a more detailed decomposition of walking behavior.

There are several strengths of the currents study such as the exploration of the impact of different window lengths and accelerometer set-ups (i.e., thigh and/or low back) on the performance of the machine learning classifier, and a large training dataset encompassing healthy adults of both genders with a wide variation in characteristics likely to influence movement pattern (i.e., age, body height, body weight, and body mass index). However, there are some limitations that should be considered when interpreting the results. First, it should be noted that the current machine learning classifier operates on periods initially detected as walking. Thus, any initial misclassification of walking adds to the misclassification of walking speed. Second, we only considered level walking/jogging and our machine learning classifier does not discriminate level walking from uphill/downhill walking or stair walking, i.e., the classifier will classify all walking as either slow, moderate, or brisk independent of whether it is performed uphill, downhill, or on level ground. Although the walking speed category is classified correctly *per se*, the associated physical exertion will be substantially higher if the walking is performed in steep uphill/downhill or in stairs. Future studies should therefore investigate whether it is possible to predict uphill/downhill walking along with walking speed *per se*. Likewise, shoe type, walking surface, carrying heavy items etc. are factors likely to influence the movement pattern and thereby the correlation between the features extracted from the acceleration signal and the walking speed. For example, walking on a soft versus firm surface will likely result in different feature characteristics, leading to a possible misclassification of walking speed category. Third, our machine learning classifier was developed based on a dataset that included healthy adults. Thus, it is uncertain if the performance of the classifier can be generalized to predict walking speed based on thigh and/or low back accelerometer data from other population groups, such as children, adolescents, older adults, or people with walking difficulties. The performance of the classifier on other population groups depends on whether the training data used to develop the classifier cover the variation in the “new” target population. Thus, if the movement patterns during different walking speeds differ substantially between the current sample of healthy adults and other target populations, the classifier will perform poorly in predicting walking speeds. Moreover, we observed considerable inter-individual variation in the prediction accuracy (e.g., 59–98 % for the dual accelerometer set-up), potentially due to differences in participant characteristics. However, our sample size was insufficient to meaningfully assess the impact of individual characteristics (e.g., age, gender, body composition, health status, preferred walking speed) on the performance of our machine learning classifier. Therefore, further studies are necessary to evaluate the generalizability of the current machine learning classifier to other populations and to determine whether individual characteristics influence the prediction accuracy. Fourth, a previous study has reported an inverse dose-dependent association between walking speeds 0.4 m/s up to 1.2 m/s and mortality among adults ≥65 years [3]. Moreover, a cut-off value for walking speed of 0.8 m/s has been suggested to identify older adults at particular high risk of frailty [35]. Thus, our cut-off for slow walking of ≤4 km/h (∼≤1.11 m/s) is too high to provide a fine-graded assessment of walking function among older adults or people with walking difficulties. Future studies are therefore necessary to develop and train machine learning classifiers that can predict lower walking speed ranges in older adults and people with walking difficulties. Recent findings suggest that slow walking speeds can be correctly classified based on electromyographic activation patterns of lower limb muscles [36]. However, whether this is possible based on accelerometer recordings alone remains unclear.

Finally, as indicated by the range of values for individual participants there was a considerable overlap in %HRmax and perceived exertion between the adjacent walking conditions and between brisk walking and jogging. This overlap is due to the interindividual differences in physical capacity, highlighting that absolute walking speed is a poor measure of the relative intensity for individuals with very low or very high physical capacity. This limitation is important to consider for studies adopting the current machine learning classifier in future studies.

## 5 Conclusions

In conclusion, a machine learning classifier can predict slow, moderate, and brisk walking speeds with high accuracy based on a single and dual accelerometer set-up (thigh and/or low back). Moreover, brisk walking can be separated from jogging with high accuracy. The training data used to develop the classifier as well as the parameters of the classifier are publicly available and can be adopted by other studies aiming to assess walking speed based on a single or dual accelerometer set-up at the thigh and/or low back.

## CRediT authorship contribution statement

**Aleksej Logacjov:** Writing – review & editing, Visualization, Validation, Software, Methodology, Investigation, Formal analysis, Data curation. **Tonje Pedersen Ludvigsen:** Writing – review & editing, Methodology, Investigation, Formal analysis, Data curation, Conceptualization. **Kerstin Bach:** Writing – review & editing, Validation, Supervision, Software, Resources, Project administration, Methodology, Investigation, Funding acquisition, Formal analysis, Data curation, Conceptualization. **Atle Kongsvold:** Writing – review & editing, Validation, Methodology, Investigation, Formal analysis, Data curation, Conceptualization. **Mats Flaaten:** Writing – review & editing, Methodology, Formal analysis. **Tom Ivar Lund Nilsen:** Writing – review & editing, Resources, Methodology, Investigation, Formal analysis, Conceptualization. **Paul Jarle Mork:** Writing – review & editing, Writing – original draft, Visualization, Validation, Supervision, Project administration, Methodology, Investigation, Funding acquisition, Formal analysis, Data curation, Conceptualization.

## Funding

This research study was supported by NTNU Health, 10.13039/100009123Norwegian University of Science and Technology (grant no. 81771516).

## Declaration of competing interest

The authors declare that they have no known competing financial interests or personal relationships that could have appeared to influence the work reported in this paper.

## Glossary

HRmax: maximum heart rate
MET: metabolic equivalents of task
XGBoost: eXtreme Gradient Boosting
