# Supplementary material for: The performance of a machine learning model in predicting accelerometer-derived walking speed
Source: Heliyon. 2025 Jan 22;11(2):e42185. doi: 10.1016/j.heliyon.2025.e42185 (PMC11804687; doi:10.1016/j.heliyon.2025.e42185)
Supplement: Multimedia component 1 [file mmc1.docx]

Supplementary Material 1. Performance metrics for detection of the three walking speeds (slow, moderate, and brisk) and jogging for the different accelerometer set-ups and window lengths. Values are mean (SD) and range for individual participants.

|  | Thigh and back | Back | Thigh |
| --- | --- | --- | --- |
| Sensitivity |  |  |  |
| 1 s windowing | 0.90 (0.09), 0.67-0.99 | 0.91 (0.08), 0.65-0.99 | 0.89 (0.10), 0.56-0.97 |
| 3 s windowing | 0.91 (0.10), 0.66-0.99 | 0.91 (0.09), 0.70-0.98 | 0.91 (0.10), 0.56-0.99 |
| 5 s windowing | 0.91 (0.10), 0.64-0.99 | 0.91 (0.09), 0.66-0.98 | 0.91 (0.10), 0.60-0.99 |
| Specificity |  |  |  |
| 1 s windowing | 0.92 (0.07), 0.72-0.99 | 0.92 (0.06), 0.72-0.99 | 0.91 (0.08), 0.66-0.97 |
| 3 s windowing | 0.92 (0.08), 0.74-0.99 | 0.92 (0.07), 0.75-0.98 | 0.92 (0.08), 0.66-0.99 |
| 5 s windowing | 0.93 (0.08), 0.72-0.99 | 0.92 (0.08), 0.69-0.98 | 0.92 (0.08), 0.70-0.99 |
| Accuracy |  |  |  |
| 1 s windowing | 0.90 (0.10), 0.63-0.99 | 0.90 (0.09), 0.62-0.99 | 0.88 (0.11), 0.51-0.96 |
| 3 s windowing | 0.90 (0.11), 0.62-0.98 | 0.91 (0.10), 0.67-0.98 | 0.90 (0.12), 0.50-0.99 |
| 5 s windowing | 0.91 (0.11), 0.59-0.98 | 0.91 (0.10), 0.63-0.98 | 0.90 (0.12), 0.55-0.99 |
| F1 score |  |  |  |
| 1 s windowing | 0.90 (0.10), 0.64-0.99 | 0.91 (0.08), 0.63-0.99 | 0.88 (0.12), 0.49-0.97 |
| 3 s windowing | 0.90 (0.11), 0.61-0.99 | 0.91 (0.10), 0.66-0.98 | 0.90 (0.12), 0.48-0.99 |
| 5 s windowing | 0.90 (0.11), 0.59-0.99 | 0.91 (0.10), 0.63-0.98 | 0.90 (0.12), 0.55-0.99 |
